# Romantic Love and Sleep Variations: Potential Proximate Mechanisms and Evolutionary Functions

**DOI:** 10.3390/biology10090923

**Published:** 2021-09-16

**Authors:** Adam Bode, Liisa Kuula

**Affiliations:** 1 School of Archaeology and Anthropology, ANU College of Arts and Social Sciences, The Australian National University, Canberra ACT 0200, Australia; 2 SleepWell Research Program, Faculty of Medicine, University of Helsinki, 00100 Helsinki, Finland; liisa.kuula@helsinki.fi

**Keywords:** romantic love, sleep, Tinbergen, mechanisms, functions, evolution

## Abstract

**Simple Summary:**

Romantic love is a topic of immense interest to scientists and the general public. This article defines and describes romantic love and sleep. It then summarises what research says about sleep in people experiencing romantic love. People in love experience changes in their sleep. We explain why people in love may experience sleep changes and what evolutionary purpose they might serve. We suggest that we are not able to determine whether sleep changes are a result of evolution or a secondary consequence. We finish by suggesting ideas for future research.

**Abstract:**

This article provides a narrative review of what is known about romantic love and sleep variations and provides possible explanations for the association. Romantic love and sleep are described using a comprehensive, unifying framework advocated by Tinbergen. We summarise the findings of studies investigating the relationship between romantic love and sleep. Sleep variations are associated with romantic love in adolescents and young adults. We then detail some proximate mechanisms that may contribute to sleep variations in people experiencing romantic love before considering potential evolutionary functions of sleep variations in people experiencing romantic love. The relationship between symptoms of psychopathology and sleep variations in people experiencing romantic love is described. With the current state of knowledge, it is not possible to determine whether sleep variations associated with romantic love are adaptations or by-products of romantic love. We conclude by proposing areas for future research.

## 1. Introduction

Romantic love is commonly associated with a change in behaviour during both day and night including variation in a range of sleep measures. This article provides a narrative review of what is known about romantic love and sleep and provides possible explanations for the association. First, we describe romantic love and sleep using a comprehensive, unifying framework advocated by the Dutch biologist, Nikolaas Tinbergen [1]. This is a framework used to investigate a biological trait using the full spectrum of approaches found in biology. Second, we summarise the findings of studies investigating the relationship between romantic love and sleep. Third, we detail some mechanisms that may contribute to sleep variations in people experiencing romantic love. We consider the role of testosterone, serotonin, dopamine and its activity in the mesolimbic pathway, oxytocin, cortisol, nerve growth factor, bed-sharing, and elevated mood in sleep variations in people experiencing romantic love. Fourth, we consider potential evolutionary functions of sleep variations in people experiencing romantic love. Fifth, we argue that, with the current state of knowledge, it is not possible to determine whether sleep variations associated with romantic love are adaptations or by-products of romantic love. We conclude by proposing areas for future research.

## 2. Biological Perspectives on Romantic Love and Sleep

This section describes romantic love and sleep using a framework advocated by Tinbergen [1]. This framework uses the full breadth of approaches found in biology to provide a comprehensive account of a biological trait. It incorporates aspects of earlier biological frameworks (e.g., [2]). Tinbergen’s “four questions”, as they are known, ask about the mechanisms that cause a trait, ontogeny of a trait (development across the lifespan), evolutionary functions (functions), and phylogeny (evolutionary history). Mechanisms and development across the lifespan are considered “proximate” explanations of a trait, whereas functions and evolutionary history are considered “ultimate” explanations of a trait [1,2,3], and they benefit from being considered together [4]. Answering one question provides complementary insights into the other questions [3]. This approach has been used to describe traits across the major kingdoms of life (e.g., [5,6,7]). It has been used to describe complex human traits including infant crying [8], prosocial bias in favour of attractive people [9], the female sexual orientation spectrum [10], and romantic love [11].

### 2.1. Romantic Love

“Romantic love is a motivational state typically associated with a desire for long-term mating with a particular individual. It occurs across the lifespan and is associated with distinctive cognitive, emotional, behavioural, social, genetic, neural, and endocrine activity in both sexes. Throughout much of the life course, it serves mate choice, courtship, sex, and pair-bonding functions. It is a suite of adaptations and by-products that arose sometime during the recent evolutionary history of humans” [11] (p. 21).

As above-mentioned, Bode and Kushnick [11] describe the mechanisms, development across the lifespan, functions, and evolutionary history of romantic love in detail. In summary, romantic love is caused by social and interactive characteristics: reciprocal liking, propinquity, social influence, and the filling of needs [12,13,14]. It is generated by psychological mate choice mechanisms: mate preferences [15], attraction [16], and sexual desire [17]. Specific genetic polymorphisms that regulate dopamine 2 receptors, vasopressin receptors, oxytocin receptors, dopamine 4 receptors, and dopamine transmission are associated with romantic love [18,19]. Romantic love is driven by activity in various neurobiological systems: mesolimbic reward pathway (e.g., ventral tegmental area, nucleus accumbens, amygdala, and medial prefrontal cortex [20]), emotion regulation (e.g., amygdala, anterior cingulate cortex, and the insula) (see [11]), sexual desire and arousal (e.g., caudate, insula, putamen, and anterior cingulate cortex) [21,22], social cognition (e.g., amygdala, insula, and medial prefrontal cortex) (see [11]), and others [23]. It is also caused by endocrinological activity in multiple systems: those that regulate sex hormones (i.e., testosterone, follicle-stimulating hormone, luteinising hormone), serotonin, dopamine, oxytocin, cortisol, and nerve growth factor [24,25,26,27,28,29,30,31]. Romantic love can first emerge in childhood [32], becomes more frequent and expresses with most of its characteristics in adolescence, but manifests throughout the lifespan [33].

Romantic love serves the evolutionary functions of mate choice [34], courtship [34], sex [35], and pair-bonding [36]. Romantic love probably evolved by co-opting mother–infant bonding sometime prior to, or following, the human line split from our common ancestor with chimpanzees and bonobos [11].

### 2.2. Sleep

#### 2.2.1. Definition, Characteristics, and Measurement

Sleep is common in the animal kingdom, although it takes various forms [37,38]. Sleep in humans is defined “on the basis of both behaviour of the person while asleep and the related physiologic changes that occur to the waking brain’s electrical rhythm in sleep” [39] (p. 7). Behavioural characteristics of sleep include lack of mobility or slight mobility, closed eyes, a characteristic species-specific sleeping posture, reduced response to external stimulation, quiescence, increased reaction time, elevated arousal threshold, impaired cognitive function, and a reversible unconscious state. It includes non-rapid eye movement (NREM) sleep and rapid eye movement (REM) sleep. Non-rapid eye movement sleep is characterised by synchronised electroencephalographic activity, mildly reduced muscle tone, and slow rolling eye movements. Rapid eye movement sleep is characterised by theta or saw tooth waves and desynchronised electroencephalographic activity, moderately to severely reduced or absent muscle tone, and rapid eye movements [39]. There are three stages of NREM sleep (i.e., N1, N2, N3) and one stage of REM sleep [40].

**Figure 1:**
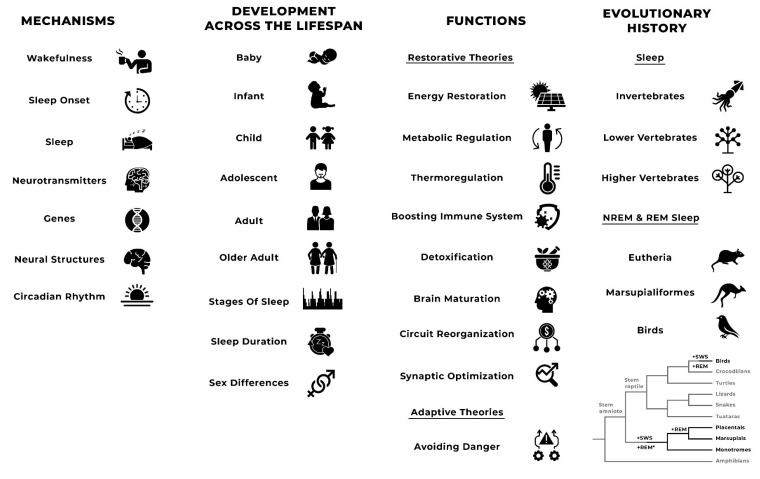
**List of features of sleep according to Tinbergen’s four questions.** Features of sleep are presented according to the order in which they are presented in this article. Cladogram taken from Lesku and colleagues [56].

Sleep can be measured in multiple ways. These can include self-report [41], observational [42], and objective methods (see [43]). Self-report measures can include the collection of data about sleep onset, sleep timing, sleep duration, wake after sleep onset (WASO), sleep quality, restoration after sleep, sleep regularity, and causes of sleep disturbance (see, for example, [41,44,45,46,47,48]). Observational methods can identify the behavioural characteristics of sleep. The two most common objective measures of sleep are the accelerometry or actigraphy, which measures movement of the individual, and polysomnography (PSG) [49]. Polysomnography is a systematic process used to collect physiologic parameters during sleep [50]. It involves a combination of electroencephalogram (EEG), electro-oculogram (EOG), electromyogram (EMG), electrocardiogram (ECG), pulse oximetry, and measures of airflow and respiratory effort. It is the only means of assessing stages of sleep and is the gold standard for sleep research and diagnosing sleep disorders. Despite PSG being the gold standard, the full range of methods may be useful in detecting different aspects of sleep. Figure 1 presents a list of the features of sleep according to Tinbergen’s [1] four questions.

#### 2.2.2. Mechanisms

Mechanisms relating to different aspects of sleep have been studied in both animals and humans (see [51,52]). Wakefulness is regulated by the basal forebrain, lateral hypothalamus, tuberomammillary nucleus, and brainstem, with involvement of norepinephrine, dopamine, serotonin, acetylcholine, histamine, hypocretin, and neuropeptide S systems (see [53] for review). Sleep onset is induced by cytokines and hormones, adenosine, prostaglandins, anandamide, and urotensin II (see [53] for review). A number of neural structures regulate sleep: the suprachiasmatic nucleus (SCN), basal forebrain, medial, lateral, and ventrolateral preoptic nuclei, and brainstem (see [53] for review). The primary neurochemical mechanisms that regulate sleep include gamma-aminobutyric acid and acetylcholine (see [53] for review). Some immediate-early genes are up- or downregulated during sleep compared to the waking state (see [53] for review). Specifically, the preoptic area, basal forebrain, and cortical sleep-active neuronal nitric oxide synthase neurons may play particularly important roles in NREM sleep (see [53] for review). The pedunculopontine/laterodorsal tegmental nuclei, sublaterodorsal nucleus, medullary reticular formation, and parts of the hypothalamus probably play specific roles in REM sleep (see [53] for review).

One of the key mechanisms that regulates the sleep–wake cycle as well as metabolism, heart rate, blood pressure, body temperature, renal activity, and hormone secretion is the circadian rhythm. The circadian rhythm results from environmental cues (i.e., light exposure) as well as an endogenous circadian timing system. This timing system throughout the body is largely regulated by two clusters of neurons in the SCN, located in the anterior hypothalamus, which coordinate overt rhythms through neuronal and hormonal outputs [54]. Another key mechanism might be the basic rest–activity cycle, which is a physiological rhythm and has a period shorter than 24 h, running throughout the 24 h with four cycles during the day and five at night [55].

#### 2.2.3. Development across the Lifespan

In the first few weeks of life, sleep can total up to 16 h in a day. By about six weeks of life, the infant is more awake during the day and sleeps more at night. By four months of age, most infants sleep most of the night. At this stage of development, infants have three distinct sleep stages: active sleep, quiet sleep, and intermediate sleep. In the first year of life, sleep duration averages 14 h in a day and by six months of age, infants are generally sleeping predominantly at night (see [57]). In early childhood, the stages of sleep are the same as in adults, although the length each one lasts is different. Prior to the onset of puberty, children sleep about 9–11 h per day, almost exclusively at night (see [57]). At puberty, both sleep onset and natural awakenings are delayed. In the transition from adolescence to adulthood, the length of various NREM stages change, resulting in lighter sleep. The delayed onset of sleep and wakening associated with puberty subsides in adulthood and a 90 min sleep cycle of NREM-to-REM stages is established with all sleep stages represented (see [57]). There is a small reduction in REM sleep in early- and mid-adulthood. Older age is associated with earlier sleep onset and poorer sleep quality (see [56]; see also [58] for summary of ontogeny of functions at specific developmental stages).

Sleep disturbances can affect any age group and are not a normal part of ageing (see [59]). However, sleep problems are common among older people because medical conditions and changes in social engagement, lifestyle, and living environment associated with ageing can contribute to sleep problems [60]. Artificial light and mistimed light, associated with the modern environment, affects both circadian rhythms and sleep–wake cycles [61,62].

There are some sex differences in human sleep. Females report poorer sleep quality and a greater risk of developing some specific sleep disorders such as insomnia than males [63,64]. Males, on the other hand, have a greater risk of developing some other sleep disorders such as obstructive sleep apnoea than females [63,64]. Variations in hormones, physical and mental condition, social roles, and ageing are among the factors that explain this sex difference. Sleep disturbances are common during menstruation, pregnancy, and menopause (see [63,65]). Later sleep timing associated with puberty starts earlier in females, because, on average, they reach puberty earlier than males.

#### 2.2.4. Functions

Two lines of theory outline the functions of sleep: restorative theories and adaptive theories [66]. Restorative theories suggest that sleep serves a number of functions including energy restoration, metabolic regulation, thermoregulation, boosting the immune system, brain and body detoxification, brain maturation, circuit reorganisation, and synaptic optimisation [67]. It is essential for many vital functions including physiological, somatic, and neuroanatomical development, energy conservation, brain waste clearance, modulation of immune responses, cognition, performance, vigilance, disease response, and psychological state [51]. Long-term sleep loss and sleep disorders have been associated with a number of deleterious health effects including cancer [68], hypertension, type 2 diabetes, obesity, depression, heart attack, and stroke [69]. Common sleep disorders involve, or include respiratory disorders of sleep, insomnia, hypersomnia, parasomnia, circadian rhythm disorders, and sleep movement disorders [70].

Non-rapid eye movement sleep is associated with immune system function by playing a role in the formation of immunological memory [71] and supporting the immune system’s ability to anticipate infectious threats from injury [72]. Slow cortical oscillations in NREM sleep facilitate restoration and repair of the body and the nervous system—the latter probably by enabling information processing, synaptic plasticity, and prophylactic cellular maintenance [73]. This also facilities memory processing. The three stages of NREM sleep are associated with a progressive deactivation of a select group of neurons in the brain structures that are reactivated during REM sleep [74]. It is for this reason that REM sleep has been dubbed “paradoxical sleep”. High levels of brain metabolic demand and attenuation of homeostatic regulation make it unclear how REM sleep can be adaptive in the broader context of sleep [75].

Adaptive theories suggest that animals sleep to avoid danger. At first glance, it appears that, during sleep, an individual is largely non-responsive to environmental stimuli, placing them at risk of harm from the social and physical environment. This is true, but it must be considered in the context of trade-offs and our evolutionary history. The result of trade-offs (see [76]) indicates that the benefits of sleep outweigh any immediate costs to survival. It should also be noted that sleep evolved long ago in our evolutionary history, and we most likely sleep at night because this is the time when resource collection is at its lowest and is the period in which the greatest risk of predation existed during our evolutionary history (see [66]). There is empirical support for the notion that a sleep strategy is adaptive in most contexts [77].

#### 2.2.5. Evolutionary History

We know relatively little about the evolution of NREM and REM sleep, or sleep generally [38]. Sleep involving NREM and REM sleep is ubiquitous among placental (*Eutheria*) and marsupial (*Marsupialiformes*) mammals and birds [56]. Sleep is common in reptiles, amphibians, and fish [39]. Behaviour analogous to sleep has been identified in numerous invertebrates and lower vertebrates [67], suggesting that it is evolutionarily old. Non-rapid eye movement sleep and REM sleep are thought to have evolved as a differentiation of a single, phylogenetically older sleep state [78]. REM sleep, or a precursor state with aspects of REM sleep, may have originated in reptiles [79,80]. The presence of both types of sleep in birds and mammals is probably the result of parallel evolution [56].

## 3. Romantic Love and Sleep Variations

To our knowledge, there are seven studies that have empirically investigated sleep in people experiencing romantic love [45,46,47,48,81,82,83]. These studies have investigated adolescents and young adult females and males in Iran, German-speaking countries, and Finland. Measures of romantic love include individual questions about love status or a variation of the Yale–Brown Obsessive Compulsive Scale [84], which measures the intensity of romantic love. Sleep is generally measured by self-report questionnaires. However, one study [83] used an accelerometer, although this was an average of 7.2 months after participants self-reported being in love. Aspects of sleep for which data have been collected include sleep onset latency, sleep duration, wake after sleep onset (WASO), sleep quality, and restoring sleep, although several other related factors have also been investigated (i.e., concentration during the day, tiredness during the day, and mood). Kuula and colleagues [83] investigated the clock times at which individuals slept. Studies used parametric and nonparametric tests to identify relationships between romantic love and sleep features.

The sum of evidence is mixed regarding the effect of romantic love on sleep. There appears to be an age-related effect; some self-reported sleep features are associated with romantic love in young adults, but not adolescents, although this could be the result of the methods employed in these studies. The two studies investigating sleep variations in young adults measured the association of romantic love intensity and sleep features, whereas the majority of studies investigating adolescents simply grouped participants according to the presence or absence of romantic love, so the intensity of romantic love would have been variable in these groups. One study [82] was a longitudinal study of adolescents that found no relationship between sleep features and either the onset or extinction of romantic love. These findings suggest that factors that influence sleep (e.g., developmental stage) may also moderate the effect of romantic love on sleep. Table 1 presents a summary of the evidence supporting the influence of particular sleep features in adolescents and young adults who were experiencing romantic love.

The evidence is mixed on the effect of romantic love on sleep onset. Sleep onset latency appears to be affected by romantic love in young adults but not adolescents. None of the studies investigating self-reported sleep onset latency in adolescents experiencing romantic love [45,46,81] found a significant difference in self-reported sleep onset latency. Falling in love and falling out of love were not associated with differences in self-reported sleep onset latency [82]. Two studies [47,48] did, however, find that a greater intensity of romantic love was associated with shorter self-reported sleep onset latency in young adults. Kuula and colleagues [83] found that female adolescents who were in love and in a relationship had the latest self-reported sleep midpoint among any group in their sample, suggesting that sleep onset may be delayed for this group.

The evidence is mixed about the effect of romantic love on sleep duration. Brand and colleagues [81] found shorter self-reported sleep duration in adolescents experiencing romantic love compared to controls and shorter sleep duration with greater intensity of romantic love. Kuula and colleagues [83] found that adolescent females experiencing romantic love self-reported shorter sleep duration than the controls. All other studies that investigated sleep duration [45,46,47,48,82] found no significant effect.

The evidence is mixed about the effect of romantic love on WASO. Wake after sleep onset appears to be reduced by romantic love in young adults but not adolescents. Studies that investigated self-reported number of WASO in adolescents found no significant effect of romantic love [45,46]. The two studies on young adults [47,48], however, found that the intensity of romantic love was negatively associated with self-reported number of bouts of WASO.

The evidence is mixed regarding the effect of romantic love on sleep quality. Brand and colleagues [81] found a significant effect of romantic love on sleep quality. In that study, adolescents who were in love reported better sleep quality than the controls (although it is important to note that the study excluded participants that might meet the criteria for a psychiatric disorder). The remaining adolescent studies [45,46,82,83] found no significant effect of romantic love on sleep quality. Both studies of young adults [47,48], however, found that the intensity of romantic love was associated with better sleep quality, as measured by the Insomnia Severity Index [85], and more restoring sleep [47,48].

### Psychopathological Symptoms Associated with Sleep Variations

Just as developmental stage may play a role in moderating the relationship between romantic love and sleep, other psychological factors may influence the effect of romantic love on sleep features. Symptoms of hypomania, depression, and anxiety appear to be associated with specific sleep variations in people experiencing romantic love. This is relevant because certain symptoms of hypomania [47,48], depression symptoms [47,48,83], and anxiety symptoms [45,47,48,82,83,86] are associated with romantic love. Sleep variations associated with these symptoms in people experiencing romantic love may simply be the consequence of psychopathology. However, we think that it is also possible that these symptoms may be caused by romantic love, and any relationship between symptoms of psychopathology and sleep variations may be indirectly caused by romantic love.

Two studies of young adults [47,48] found that specific constellations of hypomanic symptoms are associated with the intensity of romantic love, and that each of these constellations is associated with different sleep variations and other symptoms of psychopathology in young adults experiencing romantic love. Active/elated hypomania symptoms were associated with shorter sleep onset latency, shorter sleep duration, fewer WASO, better sleep quality, and increased restoring sleep. Irritable/risk-taking hypomanic symptoms were associated with longer sleep onset latency, more WASO, and worse sleep quality [47,48]. One of those studies [47] found that irritable/risk-taking symptoms were associated with shorter sleep duration.

The results of both studies in young adults [47,48] found that depressive symptoms were associated with longer sleep onset latency, shorter sleep duration, more WASO, poorer sleep quality, and decreased restoring sleep. One study in adolescents [45] found that increased depressive symptoms were associated with worse sleep quality and fewer WASO, while another [83] found that increased depressive symptoms were associated with later sleep timing, shorter sleep duration, and worse sleep quality. Sleep variations and associated tiredness and fatigue are measured in some measures of depressive symptoms (i.e., BDI; BDI-II), meaning that the association may be inflated.

Both studies in young adults [47,48] found that anxiety symptoms were also associated with longer sleep onset latency, shorter sleep duration, more WASO, and poorer sleep quality. One of these studies [48] found that anxiety was associated with decreased restoring sleep. Kuula and colleagues [83] found that anxiety symptoms were associated with later sleep timing, shorter sleep duration, and poorer sleep quality in adolescent females and males. There are numerous mechanistic similarities between romantic love, hypomania, depression, and anxiety (see [11] for review). Bajoghli and colleagues [45] found that different components of anxiety (i.e., trait anxiety and state anxiety) were associated with variations in sleep onset latency, WASO, and sleep quality in adolescents.

## 4. Potential Mechanisms Explaining Sleep Variations in People Experiencing Romantic Love

The current state of knowledge does not enable the assessment of romantic love’s effects upon most of the mechanisms involved in sleep. Some of the mechanisms that cause romantic love, however, have been identified, and it is possible to consider the role of some of these mechanisms in sleep variation. The overlap may partially account for the changes in sleep documented in people experiencing romantic love. In particular, the changes in sex hormones, serotonin, dopamine, cortisol, oxytocin, and nerve growth factor in people experiencing romantic love may cause the variations in sleep onset latency, sleep duration, WASO, sleep quality, and restoring sleep also documented in this group. Bed sharing and elevated mood are also considered. Table 2 presents the mechanisms that might cause sleep variations in people experiencing romantic love. Bolded mechanisms are considered below, but we have also speculated an additional five potential mechanisms that could explain sleep variations in people experiencing romantic love.

Testosterone levels in men increase following sleep onset. Disorders of sleep including abnormal sleep quality, abnormal sleep duration, circadian rhythm disruption, and sleep-disordered breathing, can result in a reduction in testosterone levels [87]. The evidence suggests that testosterone may modulate individual vulnerability to subjective symptoms of sleep restriction. Additionally, low testosterone may affect overall sleep quality (see [87] for review). In women, fluctuations in hormones across the menstrual cycle are associated with sleep variation [88]. There is some evidence showing that lower testosterone levels are associated with increased WASO (see [88] for review). Two studies [25,30] have identified changes in testosterone levels in people experiencing romantic love. However, testosterone concentrations changed in the opposite direction in the two sexes [25] and in the opposite direction among women across the studies [25,30]. The association between testosterone levels and sleep variation means that testosterone is one possible contributor to sleep variation in people experiencing romantic love.

Serotonin is documented in animal studies to promote wakefulness and inhibit REM sleep. However, in some circumstances, it can promote sleep (see [89] for review). Moreover, serotonin couples the SCN-derived signal to the ultradian sleep–wake cycles within the longest sleep period to consolidate the sleep–wakefulness rhythm [90]. Selective serotonin reuptake inhibitors can selectively disrupt or improve sleep depending on the type and dosage (see [91] for review). Two studies [24,27] have identified changes in serotonin levels in people experiencing romantic love. Serotonin is one possible contributor to sleep variation in people experiencing romantic love. Serotonin could influence the sleep–wake cycle or wakefulness, directly, as a result of its activity in the raphe nuclei (see [53]).

Animal studies demonstrate that dopamine-rich mesolimbic pathway structures (i.e., ventral tegmental area [VTA] and nucleus accumbens [NAc]) play a role in sleep–wake regulation. The VTA plays a role in sustained wakefulness and the NAc plays a role in regulating NREM sleep (see [92] for review). The VTA and NAc are consistently identified as playing a role in romantic love (see [20] for review) and one study [29] found circulating dopamine transporter variation in people experiencing romantic love. Another study identified a genetic polymorphism associated with dopamine activity in the VTA in newlyweds experiencing romantic love [19]. The association between dopamine and sleep as well as wakefulness means that dopamine is one possible contributor to sleep variations in people experiencing romantic love. Given the substantial activation of the mesolimbic pathway in romantic love, dopamine and the activity of the mesolimbic pathway should be considered as prime candidates for future research on sleep variations in people experiencing romantic love.

Intranasal administration of oxytocin has been shown to alter sleep architecture [93]. Specifically, long-term administration of oxytocin has been shown to reduce sleep latency, increase sleep efficiency, and increase the percentage of REM sleep episodes. Given that substantially elevated oxytocin levels are associated with the early stages of a romantic relationship [94,95,96] and oxytocin has long been theorised to play a role in romantic love (see [11,16]) oxytocin is one possible contributor to sleep variation in people experiencing romantic love.

The key stress hormone, cortisol, is one further potential pathway for disrupted sleep among those in love. Elevated evening cortisol secretion is associated with shorter sleep duration and greater sleep disturbance [97]. Three studies [25,28,31] have found variations in cortisol levels in people experiencing romantic love. These findings suggest that cortisol may be one contributor to sleep variations in people experiencing romantic love.

Animal studies demonstrate that nerve growth factor can influence both REM and NREM sleep [98,99]. Elevated levels of circulating nerve growth factor are associated with romantic love [26]. As a result, nerve growth factor concentrations should be studied in humans to detect how much it might explain sleep variations in people experiencing romantic love.

The presence of a romantic partner in an individual’s bed is associated with better subjective sleep quality, more REM sleep, and more stable REM sleep (see [100] for review; see also [101]). Even the simple exposure to a romantic partner’s scent during sleep can slightly improve sleep efficiency [102]. Bed sharing, which is probably more common among young adults than adolescents, may be one contributor to sleep variations in people experiencing romantic love, especially better sleep quality and restoring sleep. The absence of bed-sharing in adolescents may account for the relative lack of sleep variations in this group.

Elevated mood is associated with sleep variations. Dominance of positive affect is probably associated with better sleep features in healthy people [103]. A decreased need for sleep is a diagnostic criterion for hypomanic and manic episodes [104]. Active/elated hypomanic symptoms are particularly associated with shorter sleep onset latency, shorter sleep duration, fewer WASO, better sleep quality, and increased restoring sleep. Romantic love is consistently associated with elevated mood [45,46,47,48,81,82]. Increased hypomanic symptoms in romantic love could be the result of increased inputs into the behavioural activation system or increased sensitivity of the behavioural activation system (see [105]; see also [106] for detailed descriptions of behavioural activation system sensitivity in mania).

### Potential Mechanisms Explaining the Relationship between Symptoms of Psychopathology and Sleep Variations

Irritable/risk-taking symptoms of hypomania tend to be associated with longer sleep onset latency, shorter sleep duration, more WASO, worse sleep quality, and decreased restoring sleep in young adults experiencing romantic love. Irritable/risk-taking symptoms result from interactive contributions of depressed and anxious mood with elevated mood (see [107]). When elevated mood is experienced at the same time as inputs triggering depressed and anxious mood, irritable/risk-taking elevated mood is expressed. The mechanisms of action that cause sleep disturbances in people experiencing depression or anxiety may contribute to certain sleep variations in people experiencing romantic love when depressed and anxious moods are present. Potential contributors could include mechanisms associated with both depression and romantic love (i.e., serotonin, dopamine, cortisol systems; see [11]) as well as anxiety and romantic love (i.e., serotonin and cortisol systems; see [11]). Another candidate is inflammation, which produces cytokines into the circulation (see [72]).

The association of both hypomanic symptoms and depressive symptoms with sleep variations in people experiencing romantic love draws parallels with bipolar disorder to some extent. Romantic love is associated with periods of elation and periods of lovelorn depressed mood or short-term negative mood changes. Bipolar disorder is associated with episodes of elevated mood and, usually, episodes of depressed mood. Malfunctioning of the circadian clocks and sleep disturbance are important mechanisms of action contributing to bipolar disorder [108]. However, bipolar disorder is associated with sleep variation during episodes of elevated and depressed mood [109], and this suggests the mechanisms causing sleep disturbance in people with bipolar disorder may also be involved in people experiencing romantic love (see [11] for mechanistic similarities).

## 5. Potential Functions of Sleep Variations in People Experiencing Romantic Love

Shorter sleep onset latency, shorter sleep duration, fewer WASO, and better sleep quality could all feasibly serve survival or reproductive functions. Shorter sleep duration results in nocturnal wakefulness. It has been postulated that nocturnal wakefulness could protect against attacks by animals or humans [110]. In the context of a newly formed romantic relationship, a couple may find themselves sleeping in areas alone, or away from other members of their group because humans conceal mating (see [111] for theory on why this is the case). As a result, they may be at greater risk of harm from animals or other humans. It is unknown whether human ancestors in relevant phases of our evolutionary history practiced concealed mating. It is even possible that nocturnal wakefulness may help the process of mate guarding, whereby a partner’s liaisons with alternative potential mates could be limited. Shorter sleep onset latency and fewer WASO episodes could be a means of facilitating sleep onset and sleep maintenance at a time when disruptive factors may be present such as the movements of a loved one in an individual’s arms. This speculation has to be countered by the fact, however, that WASO, which would facilitate nocturnal wakefulness, is reduced in people experiencing romantic love, making a nocturnal wakefulness function less likely. While sleep variation may serve some survival or reproductive function, nocturnal wakefulness is unlikely to have played a substantial role. Better sleep quality and restoring sleep may serve obvious survival or reproductive purposes. Better sleep quality and restoring sleep may be associated with better mood, better concentration during the day, more energy, less fatigue, and improved cognitive performance. Better sleep quality could ensure the effective expression of elevated mood and sexual behaviours.

### Potential Functions of Sleep Variations Associated with Symptoms of Psychopathology

Irritable/risk-taking symptoms of hypomania, depressive symptoms, anxiety, and sleep variations are all correlated in people experiencing romantic love [47,48]. There is a theoretical explanation for this relationship that involves an interaction between thresholds for responding to possible reward and punishment in an individual [107]. At first glance, it might be hard to envisage how some of the sleep variations associated with irritable/risk-taking symptoms such as longer sleep onset latency and worse sleep quality could serve a survival or reproductive function. However, consideration of the evolutionary functions of depression and anxiety symptoms indicates there are possible reproductive or survival functions of sleep variations associated with symptoms of psychopathology.

Depression is associated with sleep variations [104] and depressive symptoms are associated with sleep variations in people experiencing romantic love [45,47,48,83]. Depressed mood (characterised by depressive symptoms) can serve the adaptive function of extinguishing goal-oriented behaviour (see [107,112]). Longer sleep onset latency, shorter sleep duration, more WASO, worse sleep quality, and decreased restoring sleep may increase tiredness and fatigue, decrease energy, and weaken the ability to concentrate during the day (see [47,48]). These symptoms may ensure that an individual is less inclined to engage in physically or cognitively demanding activities associated with goal attainment.

Anxiety disorders are associated with sleep variations [104] and anxiety symptoms are associated with sleep variations in adolescents and young adults experiencing romantic love [45,47,48,83]. The mechanisms that cause anxiety symptoms may result in some of the sleep variations associated with romantic love. Anxiety can serve the adaptive function of fine-tuning behavioural effort based on the experience of punishment and threat [107]. It can ensure that behaviour serves to avoid punishment and other negative outcomes. Like depression, longer sleep onset latency, shorter sleep duration, more WASO, worse sleep quality, and decreased restoring sleep associated with anxiety symptoms may increase tiredness and fatigue, decrease energy, and weaken the ability to concentrate during the day. These symptoms make it less likely that an individual will engage in physically or cognitively demanding activities that could lead to punishment or other physical harms.

## 6. Are Sleep Variations in People Experiencing Romantic Love Adaptations or By-Products?

Romantic love has been described as a suite of adaptations and by-products [11] (see also [113]). An adaption, in evolutionary psychology, is “an inherited and reliably developing characteristic that came into existence as a feature of a species through natural selection because it helped to directly or indirectly facilitate reproduction during the period of its evolution” ([114] (p. 535); see also [115]). An evolutionary biology approach of defining adaptation is “a phenotypic variant that results in the highest fitness among a specified set of variants in a given environment” [116] (p. 9). A by-product is a trait that evolved “not because it was selectively advantageous, but because it was inextricably linked […] to another trait that was reproductively advantageous” [117] (p. 48). Assessment of adaptation requires consideration of the proximate mechanisms (described above) and potential ultimate functions of sleep variations in people experiencing romantic love (see [1,3,4,117]). These lines of thinking can be said for an assessment of by-products [117].

Identifying possible mechanisms of action and functions of sleep variations in people experiencing romantic love, however, is not sufficient to make claims of adaptation. They are necessary, but not sufficient, components of adaptions. Instead, it is necessary to, at least, consider issues of complexity, efficiency, reliability, specificity, capability for solving adaptive problems, evolvability [114], and reproductive success [116]. This may not be possible given the limited knowledge that exists about the mechanisms that cause sleep variations in people experiencing romantic love. What can be said, however, is that even if sleep variations serve an adaptive function, it does not necessarily mean that it is one of the adaptations that constitute romantic love. It could feasibly be a downstream consequence of these adaptations, a by-product.

## 7. Limitations of Existing Research and Areas for Future Research

There are limitations of existing research into sleep variations in people experiencing romantic love. Addressing these and identifying new areas of research provides the opportunity to reveal new knowledge about the relationship between sleep and romantic love. The most pronounced limitation of existing research into romantic love and sleep is the small number of studies undertaken. Making statements about the presence or absence of sleep variation in people experiencing romantic love needs to be done cautiously, and we recognise that future research may create the need to reassess the evidence. Additionally, while we suggest that romantic love causes sleep variations and that the mechanisms we describe may play a role, we must acknowledge that the limited number of studies and the lack of any significant associations between falling in or out of love and sleep variations in the only longitudinal study to date [82] means that we cannot say with certainty that romantic love causes sleep variations. We believe that this is the case, and there is theoretical support for this notion. However, it is also possible that sleep variations could contribute to the onset of romantic love or that a third factor may influence both the onset of romantic love and sleep variations.

There are also limitations in relation to the measurement of romantic love and sleep. No study investigating romantic love and sleep have used a validated measure of romantic love. Most studies have used a self-reported dichotomous love variable. This is not ideal because individuals can mistake companionate love with romantic love (see [118]). Even the studies that used a measure of the intensity of love failed to use a validated measure. Existing research also relies on self-reports of sleep. There has only been one attempt to objectively measure features of sleep in people experiencing romantic love [83], but that study measured sleep 7.2 months after participants indicated their love status.

Research should attempt to collect objective measures of sleep in individuals contemporaneously in love. Studies should also use validated measures of romantic love. To our best knowledge, no study has investigated sleep architecture in people experiencing romantic love (e.g., [101]). Efforts should be made to promote the use of PSG, since it may help to explain sleep variations documented in people experiencing romantic love including changes in the frequency of WASO, sleep quality, and restoring sleep. Another limitation of the existing research is that there have been no studies on the mechanisms that may cause sleep variation in people experiencing romantic love. Comparison of groups according to sleep variations and romantic love status in neuroimaging studies is one option, although methods other than those traditionally provided by fMRI may be required (i.e., electroencephalogram or positron emission tomography). Measuring circulating peptides identified in this article, either related to a period of sleep, or according to sleep profile, may be useful in shedding light on the mechanisms that cause sleep variation in people experiencing romantic love. The evidence base would also benefit greatly from another longitudinal study with sufficient sample sizes to measure small effects.

## 8. Conclusions

This article reviewed what is known about romantic love in relation to sleep and provided possible explanations for the association. First, we described romantic love and sleep using a comprehensive, unifying framework advocated by Tinbergen [1]. Second, we summarised the findings of studies investigating the relationship between romantic love and sleep. Third, we detailed some mechanisms that may contribute to sleep variations in people experiencing romantic love. We considered the role of testosterone, serotonin, dopamine, and its activity in the mesolimbic pathway, oxytocin, cortisol, nerve growth factor, bed-sharing, and elevated mood in sleep variations in people experiencing romantic love. Fourth, we considered potential evolutionary functions of sleep variations in people experiencing romantic love. Fifth, we argued that, with the current state of knowledge, it is not possible to determine whether sleep variations associated with romantic love are adaptations or by-products of romantic love. Finally, we concluded by proposing areas for future research.

## Figures and Tables

**Table 1:** Sleep variations in people experiencing romantic love (evidence from at least two studies).

	Adolescents	Young Adults	Studies
Sleep onset latency	-	Shorter	[47,48]; see also [45,46,81,82]
Sleep duration	Shorter	-	[81,83] *; see also [45,46,47,48,82]
WASO	-	Fewer	[47,48]; see also [45,46]
Sleep quality	-	Better	[47,48]; see also [81] and [45,46,47,82,83]
Restoring sleep		Increased	[47,48]

**Notes.** Refs. [47,48] investigated the intensity of romantic love; restoring sleep has not been investigated in adolescents. Ref. [81] was the only study of adolescents that found a significant association with sleep quality; WASO = wake after sleep onset; * = [83] Females only; - = non-significant association.

**Table 2:** Mechanisms that might cause sleep variations in people experiencing romantic love.

(Neuro) Endocrine	Neural	Social	Psychological
Testosterone	Mesolimbic pathway	Bed sharing	Mood
**Serotonin**		Joint evening activities	Attachment anxiety
**Dopamine**		Sexual activity	Rumination
**Oxytocin**			Stress
**Cortisol**			
**NGF**			

**Note.** Factors considered in this article are bolded; NGF = nerve growth factor.
